# Short-term risk factors for a second hip fracture in a UK population

**DOI:** 10.1007/s00590-019-02412-8

**Published:** 2019-03-12

**Authors:** Hassaan Q. Sheikh, Fahad S. Hossain, Sayeed Khan, Mohammad Usman, Harish Kapoor, Adeel Aqil

**Affiliations:** 1 Leeds Teaching Hospitals, Great George St, Leeds, LS1 3EX UK; 2 Barnsley Hospital Trust, Gawber Rd, Barnsley, S75 2EP UK; 3 Bradford Teaching Hospitals, Duckworth Ln, Bradford, BD9 6RJ UK

**Keywords:** Hip fracture, Re-fracture, Second fracture, Epidemiology, Proximal femur fracture, Osteoporosis, Risk factors

## Abstract

**Introduction:**

A hip fracture carries significant morbidity and mortality—a second fracture of the contralateral hip carries even higher complications. Most second hip fractures occur within 48 months of the first. The aim of this study was to comprehensively analyse all identifiable variables that may increase the risk of a contralateral hip fracture within this time period.

**Methods:**

We retrospectively analysed 1242 consecutive patients with hip fractures presenting to our institution. All patient-related, surgery-related and inpatient variables were collected from the index admission. We then identified patients with a subsequent contralateral hip fracture in the following 2 years. Univariate and multivariate analyses were performed to identify risk factors associated with a second fracture.

**Results:**

A total of 66 patients (5.3%) had a contralateral hip fracture in the 2 years following initial hip fracture. Mean age at first presentation was 81 years, and mean time to second fracture was 305 days. Following multivariate analysis, the patients at highest risk of a second fracture were those with dementia, acute inpatient chest infection, urinary tract infection and multiple comorbidities as measured by the Charlson score. Discharge destination after initial fracture was not associated with the risk of a second fracture.

**Conclusions:**

We have identified a number of discrete risk factors that are associated with a short- to medium-term risk of contralateral hip fracture that may be useful in screening for patients at risk and provide them with focused medical rehabilitation.

## Introduction

Hip fractures are of the commonest sequelae of osteoporosis with significantly poor patient outcomes and a massive cost burden to healthcare institutions [1]. Furthermore, they are associated with high mortality rates and development of secondary complications [2, 3]. Consequently, there is plenty of literature surrounding the epidemiology and complications of hip fractures. There are, however, fewer studies focusing on the patient population with a contralateral hip fracture following an initial hip fracture. Patients sustaining a first hip fracture are at 11–15% increased 10-year risk of second hip fracture [4]. They also have 2.5 times increased risk of other types of fracture [5]. Following a second hip fracture, patients are less likely to maintain their walking ability and social independence. Their mortality is also significantly higher, and they are likely to be older, in full-time care and suffer from dementia [6, 7]. It would be prudent, therefore, to identify and target modifiable risk factors that may increase the risk of a second hip fracture to reduce its incidence.

Population level data from Denmark suggest that associated risk factors are female gender, increasing age, excess alcohol consumption, living alone and any previous fracture [8]. Similar findings have been reported in Japan, Taiwan and USA along with additional risk factors including neurological disease, vision disturbances and inability to walk [9–11]. Registry level data, which these studies are generally based on, bring its own limitations such as high error rates and the lack of specific inpatient data, e.g. admission blood test results.

Of the measures used to protect against a second hip fracture is the hip protector—a randomised controlled trial of these in community-dwelling patients showed that they confer no benefit in reducing the incidence of a second hip fracture [12]. The only intervention that has shown to of benefit is the use of anti-osteoporotic treatment, in particular bisphosphonates [9, 13]. However, these are sometimes not prescribed after the index fracture and patients often do not comply with the treatment regularly [10, 14].

There is currently little data available from the UK surrounding the risk factors relating to a second hip fracture. Additionally, the risk factors that previous authors identified have pertained to long-term second hip fracture incidence (up to 10 years). As such, these studies have generally reported chronic, non-modifiable risk factors. It has been shown that up to 75% of second hip fractures occur within 48 months of the index fracture [15, 16]. We therefore studied the incidence and risk factors pertaining to a second, contralateral hip fracture in a UK population with the aim to identify acute risk factors that may be modifiable. The intention was that these risk factors may be identified at the index admission to allow extra resources to be focused on those patients with elevated risk.

## Methods

We retrospectively analysed our institution’s prospectively collected National Hip Fracture Database (NHFD, England and Wales) data over a 3-year period between September 2008 and March 2011 following approval by our institutional review board. This identified 1361 consecutive patients with hip fractures admitted to our unit (a large teaching hospital). Data collected included patient demographics (age, gender, admission source and walking ability), all admission blood test results and medical comorbidities, The American Society of Anesthesiologists (ASA) grade, premorbid walking ability and fracture type. All comorbidities were collected from inpatient coded databases as International Classification of Disease, 10th revision (ICD-10) codes and were used to calculate the Charlson Comorbidity Index [17]. Other data collected included time to surgery, mortality data, length of inpatient stay and discharge destination.

The NHFD was searched again to identify patients who subsequently had a contralateral fracture in the following 2 years. Patients who died within 2 years of their initial hip fracture were included in the analysis as the intention was to analyse the risk of second hip fracture within 2 years (without bias to mortality). Patients excluded were those with duplicate or incomplete records. Imaging was reviewed by two reviewers to ensure that patients with pathological fractures, ipsilateral fractures (i.e. periprosthetic fractures) and simultaneous bilateral fractures, and patients with previous implant surgery in the contralateral hip or femur were also excluded. This left a total of 1242 patients out of a total of 1361 initially identified.

All patients were treated on a standardised hip fracture pathway based on national guidelines. On presentation, all patients received full clinical assessment including physical examination, ECG, chest X-ray and blood tests including haematological, biochemical and clotting profiling. Our unit aimed to perform operative fixation of the fractures on the day of presentation or the day after. The surgical procedure for each patient was decided after review of the patient history and imaging at a multidisciplinary team meeting which included multiple orthopaedic surgeons and the anaesthetic team. All procedures were performed under laminar flow with direct supervision of a consultant trauma and orthopaedic surgeon. The majority of displaced intracapsular fractures were treated with a cemented hip hemiarthroplasty and undisplaced intracapsular fractures received in situ screw fixation. Intertrochanteric fractures were treated with a dynamic hip screw, and those with subtrochanteric extension or reverse oblique patterns were fixed with a cephalomedullary device. Patients that were not cognitively impaired and mobilised independently or with the use of a single stick received total hip arthroplasty in line with national guidance. After the procedure, all patients were mobilised immediately or the day after with physiotherapy support with the aim to fully weight bear on the affected leg.

The cohort was divided into those who sustained a contralateral fracture and those who did not. We then analysed for differences between the two cohorts in terms of the variables collected (as above) using Chi-squared/Fisher’s exact tests for categorical data and independent t test for continuous data. In this univariate analysis, *p* values of less than 0.05 were assumed to carry statistical significance. The impact of significant variables was then calculated in a multivariate regression model using Cox regression modelling with a second fracture as an endpoint. Covariates were included in the multivariate analysis if the univariate analysis resulted in a *p* value of < 0.15, in accordance with published statistical methods [18]. All statistical calculations were performed using IBM SPSS software, version 21 (Armonk, New York, USA).

## Results

The final population of 1242 patients analysed included 344 males and 898 females with a mean age at first presentation of 81 years and the mean time to the second fracture of 305 days. In total, 470 (37.8%) patients died within 2 years from the initial hip fracture—of these, 20 patients had a contralateral hip fracture. A total of 16 patients (1.3%) had a contralateral hip fracture within 90 days, 41 patients (3.3%) within 1 year and 66 patients (5.3%) within 2 years following initial hip fracture. The overall mean time to surgery was 77 h from presentation to the emergency department, and the mean length of stay was 24 days. These data are summarised in Table 1. The comorbidity data for the two groups are summarised in Table 2.

**Table 1:** Patient demographics

	Second hip fracture *n* = 66	No second hip fracture *n* = 1176	*p* value
Age	83	81	0.058
Sex			0.047
Male	12 (18.2%)	332 (28.2%)	
Female	54 (81.8%)	844 (71.8)	
Fracture type			0.181
IC—undisplaced	19 (28.8%)	369 (31.4%)	
IC—displaced	16 (24.2%)	365 (31.0%)	
IT	17 (25.8%)	288 (24.5%)	
Subtrochanteric	10 (15.2%)	69 (5.9%)	
Other	4 (6.1%)	85 (7.2%)	
Pre-op Hb (g/dL)	12.2	12.0	0.276
Pre-op WCC (X10^9^/L)	10.8	10.8	0.962
Pre-op platelets (X10^9^/L)	316	283	0.024
Pre-op Na^+^ (mmol/L)	136	137	0.026
Pre-op K^+^ (mmol/L)	4.4	4.4	0.857
Pre-op urea (mmol/L)	8.1	8.4	0.559
Pre-op creatinine (µmol/l)	96	105	0.0147
Pre-op INR	1.1	1.2	0.348
Pre-op APTT (s)	31.4	30.5	0.377
Mean time to surgery (h)	59	78	0.583
Admission from			0.377
Own home	49 (74.2%)	908 (77.2%)	
Residential/nursing home	10 (15.2%)	204 (17.3%)	
Inpatient	3 (4.5%)	30 (2.6%)	
Tertiary referral	0 (0.0%)	4 (0.3%)	
Unknown	4 (6.1%)	30 (2.6%)	
Walking ability			0.009
Independent	32 (48.5%)	649 (55.2%)	
Single aid	13 (19.7%)	283 (24.1%)	
Two aids/frame	15 (22.7%)	193 (16.4%)	
Wheelchair/electric buggy	0 (0.0%)	22 (1.9%)	
Unknown	6 (9.1%)	29 (2.5%)	
ASA			0.722
1	4 (6.1%)	108 (9.2%)	
2	16 (24.2%)	321 (27.3%)	
3	39 (59.1%)	595 (50.6%)	
4	7 (10.6%)	151 (12.8%)	
5	0 (0.0%)	1 (0.1%)	
Discharge destination			0.021
Own home/sheltered accommodation	23 (34.8%)	520 (44.2%)	
Rehabilitation unit	18 (27.2%)	247 (21.0%)	
Residential/nursing home	22 (33.3%)	268 (22.8%)	
Acute hospital	3 (4.5%)	37 (3.1%)	
Died as inpatient	0 (0.0%)	104 (8.8%)	
Mean length of stay (days)	24	24	0.935

**Table 2:** Analysis of variables between groups

	Second hip fracture *n* = 66	No second hip fracture *n* = 1176	*p* value
Alcohol excess	2	38	0.642
Hypertension	3	36	0.343
Concurrent chest infection	14	130	0.015
Concurrent urinary tract infection	17	180	0.023
Previous myocardial infarction	2	31	0.532
Chronic heart failure	30	5	0.034
Peripheral vascular disease	0	19	0.352
Cerebrovascular disease	3	44	0.461
Dementia	11	80	0.007
Chronic obstructive airways disease	9	180	0.438
Connective tissue disorder	0	0	1.000
Peptic ulcer disease	0	8	0.645
Diabetes mellitus	5	122	0.315
Chronic kidney disease	6	52	0.147
Hemiplegia	2	14	0.207
Leukaemia	0	0	1.000
Lymphoma	0	0	1.000
Solid organ malignancy	7	113	0.458
Liver disease	0	5	0.761
Mean Charlson comorbidity score	4.9	4.5	0.015

Analysis of the subgroup of 66 patients who had a second hip fracture revealed that in 59.1% of cases the fracture types were same in both hips (Table 3). Additionally, the walking ability data showed that the preinjury walking ability worsened in 24 patients, stayed the same in 17 patients and improved in 7 patients. The data on walking ability were unknown for 18 patients prior to one or both fractures.

**Table 3:** Fracture types between groups

	Fracture 2—intracapsular	Fracture 2—extracapsular	Fracture 2—other
Fracture 1—intracapsular	27	5	3
Fracture 2—extracapsular	15	12	0
Fracture 2—other	2	1	1

On univariate analysis, the patients that suffered a contralateral hip fracture were more likely to be of female gender, have a higher admission platelet count and lower serum sodium and creatinine levels. There was also an association with a poorer walking ability on admission and discharge destination other than own home/sheltered accommodation. Patients that suffered a contralateral hip fracture were more likely to suffer from acute chest infections (during the initial stay), urinary tract infections, chronic heart failure, dementia and have a higher Charlson comorbidity score.

Following multivariate analysis, the risk factors significantly associated with an increased fracture risk were chest infection during the index admission, urinary tract infections, dementia and increasing comorbidities as calculated by the Charlson score. These results are summarised in Table 4.

**Table 4:** Multivariate analysis of risk factors for second fracture within 48 months

Variable (relating to index admission)	*p* value	Hazard ratio	Confidence interval (95%)	
			Lower	Upper
Preinjury walking ability	0.063	1.219	0.989	1.504
Acute chest infection	0.037	1.907	1.038	3.503
Urinary tract infection	0.029	1.858	1.065	3.242
Increasing Charlson score	0.049	1.229	1.001	1.510
Dementia	0.028	2.163	1.087	4.304

## Discussion

Patients at significantly increased risk of a second hip fracture within 48 months were likely to have suffered from a chest infection, urine infections, have dementia and increasing comorbidities compared to patients that did not. Previous studies have also highlighted increasing age and female gender as risk factors associated with a second hip fracture [8, 19]. We did not find this in our cohort likely due to focusing on the short-term risk (within 48 months) of a second hip fracture, i.e. there was not enough time between the two fractures to show a significant age difference. However, focusing on short-term second fracture risk has allowed us to identify risk factors that are potentially modifiable from the index admission.

We found that the majority of second hip fractures were in the same location as the index fracture which is consistent with other studies [6, 15]. It has been theorised that this may be due to femoral neck shape and/or gait patterns; however, this remains unproven [20, 21]. Our results also show that in the second fracture group, mobility following the initial hip fracture worsened in half of the patients (where these data were available). Poor mobility following first hip fracture is a risk factor for a second fracture and correlates with poor levels of social independence after discharge [7, 13].

One-year risk of second hip fracture varies between 2 and 15% in the reported literature [4, 13]. Our 1-year incidence of second hip fracture was 3.3%, and 2-year incidence of 5.3% is lower than Danish and Finnish data but higher than US data [8, 13, 22]. This is likely due to regional variations in healthcare provision and life expectancies. Our incidence data compare favourably with the published literature. Nevertheless, these figures can be improved and consequently it is prudent to identify modifiable risk factors that may help lower re-fracture incidence with studies such as ours. Our study is the first to report risk factors for a second fracture in a UK population.

Dementia was the most significant risk factor in our cohort in predicting a second hip fracture, which is in agreement with previous studies [13, 23]. Dementia is a known risk factor for a first *and* subsequent hip fracture [24]. It may be suggested that patients suffering from dementia should preferentially be discharged to institutions with full-time care. However, our data did not show any association between discharge location and risk of second fracture.

A significantly higher proportion of patients in the second hip fracture group were discharged to their own home on univariate analysis. However, this effect was not significant following multivariate analysis. Other studies have shown that those with a second hip fracture are likely to be institutionalised [6]. As such, being institutionalised is likely a surrogate marker of frailty and comorbidity and therefore second fracture.

Our univariate analysis also showed that the mobility status of the group with a second hip fracture was better than those without a second fracture. This effect was not significant following multivariate analysis. Multiple authors have previously reported an increased risk of second fracture with impaired mobility [6, 13]. One paper, however, reported an increased second fracture risk with a higher functional status as well as a non-significant increase associated with poor mobility [19]. The likely explanation for this is that patients with poor mobility are likely to have lower bone mineral density and therefore be at risk of fracture. Equally, those that are more independently active are at a higher risk of falls.

A previous study found chronic respiratory disease to be associated with a second hip fracture [23]. The respiratory disease group in that study primarily suffered from chronic obstructive pulmonary disease and asthma as opposed to acute bronchial pneumonia. Their results may be explained by the fact that patients with chronic respiratory conditions are likely to have impaired mobility and may have been treated with long-term corticosteroids systemically, both of which predispose to reduced bone mineral density. Our results are novel in that we focused on acute respiratory infections (separate to chronic respiratory disease) and acute urinary tract infections and found associations with a second fracture. We postulate that both of these acute infections can increase the risk of falls by impairing balance. Additionally, the risk of a second fracture increased with increasing comorbidity as indicated by an increasing Charlson score. We therefore advise that patients with these diagnoses or a high comorbidity count receive full medical optimisation and falls risk assessment prior to discharge.

Bisphosphonate use has been proven beneficial in reducing the risk of a second fracture by previous authors [9, 25]. All patients treated for a hip fracture at our centre are assessed by orthogeriatricians and commenced on anti-osteoporotic therapy including bisphosphonates at index admission if they are not already taking these. However, compliance with bisphosphonates has shown to be low in the hip fracture cohort after discharge and the same is likely true for our cohort [14]. Further strategies need to be developed to improve compliance with medical therapy in this cohort in order to reduce the risk of a second fracture.

Our study is strengthened by the fact that we used local hospital inpatient data as opposed to registry level data which can be open to bias and errors. We are therefore confident in the robustness of our data and our findings. We also excluded patients with previous implant surgery in the contralateral hip and pathological/concomitant bilateral fractures by reviewing all the imaging. Again, this is something that registry level studies are often unable to do [8]. Our data are from a large UK teaching hospital with best care practices based on national guidelines. We are therefore assured that our results are valid for the wider UK population as well as worldwide. Importantly, we identified risk factors that are potentially identifiable and modifiable at index admission and may reduce the risk of a second fracture.

We acknowledge the limitations of this study in that it was a single-centre study and therefore lacks the numbers found in registry level data. This study design, however, did allow us to collect additional patient variables including individual comorbidities and blood test results as well as data pertaining to surgery (e.g. time to surgical intervention from admission). We chose to limit follow-up of patients to 2 years as the majority of second hip fractures have been shown to be within 48 months as opposed to a longer follow-up to allow us to identify more acute risk factors. We also did not focus on mortality and morbidity following the second fracture, but this is something well documented already in the literature.

In conclusion, we found that patients with a hip fracture are at increased risk of a second hip fracture if they suffer from dementia, chest and/or urine infections and increasing comorbidity. We recommend thorough medical optimisation of these patients and falls prevention assessment prior to discharge to an appropriate destination to reduce the risk of a second fracture.
